# A survey of cancer care institutions in Nepal to inform design of a pain management mobile application

**DOI:** 10.1186/s12904-021-00824-0

**Published:** 2021-11-05

**Authors:** Virginia LeBaron, Abish Adhikari, Rachel Bennett, Sandhya Chapagain Acharya, Manita Dhakal, Catherine E. Elmore, Kara Fitzgibbon, Rajesh Gongal, Regina Kattel, Ganesh Koirala, Martha Maurer, Daniel Munday, Bijay Neupane, Krishna Sagar Sharma, Ramila Shilpakar, Sudip Shrestha, Usha Thapa, Hexuan Zhang, Rebecca Dillingham, Bishnu Dutta Paudel

**Affiliations:** 1 University of Virginia School of Nursing, 225 Jeannette Lancaster Way, Charlottesville, VA 22908 USA; 2 Kathmandu Cancer Center, Tathali, Bhaktapur, Nepal; 3 National Academy of Medical Sciences, Bir Hospital, Kathmandu, Nepal; 4 B.P. Koirala Memorial Cancer Hospital, Chitwan, Nepal; 5 University of Virginia Center for Survey Research, Charlottesville, VA USA; 6 Hospice Nepal, Kathmandu, Nepal; 7 Nepal Cancer Hospital & Research Center, Kathmandu, Nepal; 8 University of Wisconsin School of Pharmacy, Sonderegger Research Center, Madison, WI USA; 9 Usher Institute, University of Edinburgh, Edinburgh, UK; 10 University of Virginia Center for Global Health Equity, Charlottesville, VA USA

**Keywords:** Palliative care, Cancer, Mobile health, Pain, Pain management guidelines, Nepal, Survey

## Abstract

**Background:**

One way to improve the delivery of oncology palliative care in low and middle-income countries (LMICs) is to leverage mobile technology to support healthcare providers in implementing pain management guidelines (PMG). However, PMG are often developed in higher-resourced settings and may not be appropriate for the resource and cultural context of LMICs.

**Objectives:**

This research represents a collaboration between the University of Virginia and the Nepalese Association of Palliative Care (NAPCare) to design a mobile health application (‘app’) to scale-up implementation of existing locally developed PMG.

**Methods:**

We conducted a cross-sectional survey of clinicians within Nepal to inform design of the app. Questions focused on knowledge, beliefs, and confidence in managing cancer pain; barriers to cancer pain management; awareness and use of the NAPCare PMG; barriers to smart phone use and desired features of a mobile app.

**Findings:**

Surveys were completed by 97 palliative care and/or oncology healthcare providers from four diverse cancer care institutions in Nepal. 49.5% (*n* = 48) had training in palliative care/cancer pain management and the majority (63.9%, *n* = 62) reported high confidence levels (scores of 8 or higher/10) in managing cancer pain. Highest ranked barriers to cancer pain management included those at the country/cultural level, such as nursing and medical school curricula lacking adequate content about palliative care and pain management, and patients who live in rural areas experiencing difficulty accessing healthcare services (overall mean = 6.36/10). Most nurses and physicians use an Android Smart Phone (82%, *n* = 74), had heard of the NAPCare PMG (96%, *n* = 88), and reported frequent use of apps to provide clinical care (mean = 6.38/10, *n* = 92). Key barriers to smart phone use differed by discipline, with nurses reporting greater concerns related to cost of data access (70%, *n* = 45) and being prohibited from using a mobile phone at work (61%; *n* = 39).

**Conclusions:**

Smart phone apps can help implement PMG and support healthcare providers in managing cancer pain in Nepal and similar settings. However, such tools must be designed to be culturally and contextually congruent and address perceived barriers to pain management and app use.

## Introduction

High quality palliative care, which emphasizes pain management, can greatly reduce global suffering from non-communicable diseases, such as cancer [1–6]. This is particularly important in low and middle-income countries (LMICs), where the cancer burden is rapidly growing, and where over 70% of patients are first diagnosed with late stage cancer [7–11]. Opioid medications, particularly morphine, remain the World Health Organization (WHO) gold standard to treat serious cancer pain [12]. However, in many LMICs access to opioids is limited, or absent [1, 7, 8, 13–15]. Even in LMICs with improved opioid availability, such as Nepal [16, 17], the benefit is limited if the medicines do not reach patients in need due to cultural, knowledge, or logistical barriers [18, 19]. Barriers to cancer pain management are multifactorial and can involve obstacles at the provider, patient, institutional and/or country/system level [9, 18–24].

Professional oncology organizations have created pain management guidelines (PMG) to help health care providers effectively manage pain [25–29]. Unfortunately, adherence to PMG remains low [30–33], and understanding contextual barriers to low adherence of PMG is limited, especially in LMICs [32, 34]. One challenge is that cancer care guidelines are often ‘imported’ from higher-resource, Western-oriented settings and may not translate well to LMIC settings [28, 35, 36]. To address these challenges, it is critical that guidelines are designed and created that are relevant for the LMIC context. Once culturally and contextually relevant guidelines are created, leveraging Mobile Health (‘mHealth’) – the use of mobile/wireless technology to improve healthcare – may enhance PMG implementation and adherence [37, 38].

Since its establishment in 2009, the Nepalese Association of Palliative Care [39] (NAPCare) has worked to improve opioid availability and the delivery of palliative care [16, 40–42] A key NAPCare initiative was to create Palliative Care PMG [43], based on WHO standards and adapted to the Nepal context. The NAPCare PMG are currently paper-based which has limited implementation. This research represents a global collaboration to develop a mHealth solution to increase uptake of the PMG and concurrently strengthen research capacity within Nepal.

## Methods

### Overall design

A cross-sectional survey was conducted with healthcare providers at four cancer care institutions within Nepal to inform design of a NAPCare PMG mobile app. The study was approved by the University of Virginia (UVA) Institutional Review Board and the Nepal Health Research Council and adhered to all relevant guidelines and regulations. It is important to note that methodological decisions for this study were guided, in part, by the intent of the funding mechanism to strengthen research capacity within Nepal, and to facilitate learning and mentoring opportunities between the University of Virginia (UVA) and Nepal interdisciplinary teams.

### Survey development

Our 36-item survey was collaboratively developed by the Nepal and UVA research teams, in consultation with the UVA Center for Survey Research. The survey took approximately 7 months to design (September 2018 – April 2019) and was a highly iterative and collaborative process between the Nepal and UVA research teams, working both remotely and during in-person fieldwork visits. Survey items were informed by the study aims and the broader literature related to known barriers to cancer pain management and challenges related to opioid availability in low and middle-income countries across various levels of the Social Ecological Model [44] (individual, interpersonal, institutional and country/cultural). Content validity was determined both by: 1) expert review by research team members with palliative care/oncology knowledge (4 nurses, 7 physicians, 1 social worker); and 2) qualitative pre-testing with members of the Nepalese Association of Palliative Care (NAPCare) not directly affiliated with the project (3 nurses, 5 physicians) to assess content, clarity and length of time to complete [45]. After pre-testing, survey items were revised based on feedback and the survey was finalized.

Survey items were written in English based on the Nepal team’s assessment that: 1) many terms did not have precise Nepali equivalents; 2) Nepali healthcare providers are trained in English and a high degree of English fluency was expected from respondents; and 3) the surveys would be administered face-to-face with a Nepali research team member available to assist with any translation needs. Particular attention was paid to be sure items were clear and contextually and culturally congruent/relevant. Questions were primarily quantitative (e.g., yes/no or Likert scale), with a few optional write-in responses if a participant wanted to expand upon a response and organized in four sections: 1) participant demographics; 2) knowledge, beliefs and perceived barriers related to cancer pain management; 3) awareness and use of the NAPCare PMG; and 4) smart phone use, barriers, and desired features of a mobile app.

### Sample and setting

We recruited a quota sample of healthcare providers over age 18 who provide direct care to patients with cancer. The target number of respondents for each site was determined by the size of the institution (for example, the hospice facility operates with a small staff of approximately 10 healthcare providers and thus had a target of 7 providers) and took into account what Nepal research team members felt were realistic recruitment goals. Potential respondents were identified by Nepal research team members, screened for eligibility, and offered a chance to participate in the survey. All participants provided verbal informed consent, which was approved by respective ethical committees (e.g., Social and Behavioral Sciences Institutional Review Board and Nepal Health Research Council), prior to any data collection. Participants reviewed an approved study information document and had the opportunity to discuss any questions with a trained research team member before proceeding with the survey. The sample was stratified across a public general hospital; a public cancer hospital; a private cancer hospital; and a hospice within Kathmandu Valley and Chitwan District, Nepal. The 4 study sites were selected because they see a high volume of cancer patients and represent diverse practice contexts.

### Data collection procedures

Pencil-and-paper surveys were administered to consented participants between May – August 2019 by trained Nepali research team members. We used paper surveys due to factors related to cost, convenience and flexibility in the field. Participants received a small cash incentive for completing the survey (local equivalent of approximately $10 USD). Completed survey data were entered into Qualtrics for data storage and management. We elected to use Qualtrics for initial data entry due to ease of user interface and to familiarize the Nepal team with the Qualtrics platform.

### Data preparation and analysis

Qualtrics survey data were exported into SPSS v.26.1 (and later to GNU PSPP 1.0.1, an open-access data analysis program similar to SPSS and more readily available to Nepal team members). Data analysis was conducted both at UVA and by members of the Nepal team, with mentorship and support from researchers at UVA.

During a fieldwork trip to Nepal in August 2019, the UVA and Nepal team conducted a thorough data audit, which included: 1) visual inspection of all paper surveys and cross-checking study identification numbers with the master participant log from each site; and 2) validating data entered into SPSS with paper survey responses with a random sample of at least 15% of surveys from each site. If data entry errors were discovered, we performed additional random validity checks to ensure accurate data entry of that site. Also, if two answers were marked on an item that only allowed for one response – and it was difficult/impossible to know which response the participant truly intended – then we used a coin toss to determine the response. We also reviewed all open text items and ‘other’ write-in options across the entire data set to ensure responses were correctly captured in SPSS; we made slight corrections for clarity and/or completeness.

Descriptive statistics were calculated for all quantitative items. Additionally, inferential comparative analyses were run to explore how variables of interest assessed by the survey (such as confidence in pain management tasks) may vary by institution (public general hospital; private cancer hospital; public cancer hospital; hospice) and healthcare provider role (nurse, physician). Specifically, Pearson chi-squared tests and independent sample t-tests were conducted to check for statistically significant relationships (α = 0.05). Our survey measures aimed to understand perceptions and experiences of respondents across individual survey items rather than present composite scales or indices. However, for the grouped personal, institutional, and country/cultural barriers to pain management, we did calculate Cronbach’s Alpha and all had values above 0.8, (individual barriers, 0.945; institutional barriers, 0.945; country/cultural barriers, 0.843) indicating strong internal reliability.

Most open text responses were brief, one phrase responses (e.g., for the write-in question, ‘do you use other guidelines or protocols to help manage cancer pain?’ the most common response was simply ‘WHO’) and were therefore simply counted, quantified, and presented with corresponding quantitative items. The last survey item, ‘is there anything else you would like to share about your experience with cancer pain in Nepal?’ yielded more detailed text responses which were exported into Microsoft Word, organized into groups based on similarity of key phrases, and then reviewed for patterns using a basic qualitative descriptive approach [46]. For example, many responses mentioned the difficulty of receiving palliative care in rural areas; these responses were grouped together under a category labeled ‘access to care.’

## Results

With the exception of Tables 1 (demographics) and 5 (opioid availability) results focus on nurse and physician respondents (*n* = 92), as they are expected to be the primary mobile app users. Findings are presented below by survey section.

### Section 1: demographics: Table 1

A total of 97 healthcare providers (64 nurses; 28 physicians; 2 pharmacists; 3 students/trainees) completed the survey across all four study sites. Overall, the majority of respondents were nurses (*n* = 64; 66%), female (*n* = 72; 74%), identified palliative care as their current practice area (*n* = 57; 59%), and reported spending over 80% of their time caring for patients with cancer (*n* = 49; 51%).

Almost equal numbers completed formal training in palliative care or cancer pain management (*n* = 48; 49%) compared to those who did not (*n* = 49; 51%). Overall, a higher percentage of nurses completed training (58%, *n* = 37) compared to physicians (39%, *n* = 11) and nurses rated its helpfulness (0, not helpful to 10, very helpful) as higher than physicians (nurses’ mean 8.7, *n* = 37; physicians’ mean 7.5, *n* = 11). This difference was statistically significant in the overall sample and within the public general hospital.

**Table 1:** Demographic characteristics of survey sample, overall, by institution and by role

	Overall				Public General Hospital			Public Cancer Hospital		Private Cancer Hospital			Hospice
	100% (97)				31% (30)			31% (30)		31% (30)			7.2% (7)
Roles *% within row total(n)*	Total	Nurse	Physician	Other	Nurse	Physician	Other	Nurse	Physician	Nurse	Physician	Other	Nurse
	100%(97)	66%(64)	29%(28)	5%(5)	70%(21)	20%(6)	10%(3)	47%(14)	53%(16)	73%(22)	29%(6)	7%(2)	100%(7)
*Time at institution (% within column total(n))*													
<1 year	20%(19)	**19%(12)**	**25%(7)**	**0%(0)**	24%(5)	33%(2)	0%(0)	14%(2)	6%(1)	**5%(1)**	**67%(4)**	**0%(0)**	57%(4)
1-5 years	43%(42)	**52%(33)**	**14%(4)**	**100%(5)**	57%(12)	33%(2)	100%(3)	0%(0)	0%(0)	**96%(21)**	**33%(2)**	**100%(2)**	0%(0)
6-10 years	11%(11)	**8%(5)**	**21%(6)**	**0%(0)**	5%(1)	0%(0)	0%(0)	21%(3)	38%(6)	**0%(0)**	**0%(0)**	**0%(0)**	14%(1)
>10 years	26%(25)	**22%(14)**	**39%(11)**	**0%(0)**	14%(3)	33%(2)	0%(0)	64%(9)	56%(9)	**0%(0)**	**0%(0)**	**0%(0)**	29%(2)
*Age Band (% within column total(n))*													
18 – 30 years	52%(50)	**66%(42)**	**18%(5)**	**60%(3)**	67%(14)	17%(1)	67%(2)	36%(5)	6%(1)	86%(19)	50%(3)	50%(1)	57%(4)
31 – 40 years	23%(22)	**16%(10)**	**36%(10)**	**40%(2)**	14%(3)	50%(3)	33%(1)	29%(4)	31%(5)	14%(3)	33%(2)	50%(1)	0%(0)
41 – 50 years	24%(23)	**19%(12)**	**39%(11)**	**0%(0)**	19%(4)	33%(2)	0%(0)	36%(5)	50%(8)	0%(0)	17%(1)	0%(0)	43%(3)
51 – 60 years	2%(2)	**0%(0)**	**7%(2)**	**0%(0)**	0%(0)	0%(0)	0%(0)	0%(0)	13%(2)	0%(0)	0%(0)	0%(0)	0%(0)
> 60 years	0%(0)	**0%(0)**	**0%(0)**	**0%(0)**	0%(0)	0%(0)	0%(0)	0%(0)	0%(0)	0%(0)	0%(0)	0%(0)	0%(0)
*Gender (% within column total(n))*													
Female	74%(72)	**98%(63)**	**25%(7)**	**40%(2)**	**100%(21)**	**17%(1)**	**33%(1)**	**100%(14)**	**19%(3)**	**100%(22)**	**50%(3)**	**50%(1)**	86%(6)
Male	26%(25)	**2%(1)**	**75%(21)**	**60%(3)**	**0%(0)**	**83%(5)**	**67%(2)**	**0%(0)**	**81%(13)**	**0%(0)**	**50%(3)**	**50%(1)**	14%(1)
*Total years’ experience: Any practice area (% within column total(n))*													
<1 year	9%(9)	**11%(1)**	**7%(2)**	**0%(0)**	10%(2)	0%(0)	0%(0)	7%(1)	0%(0)	5%(1)	33%(2)	0%(0)	43%(3)
1-5 years	31%(30)	**31%(20)**	**18%(5)**	**100%(5)**	48%(10)	50%(3)	100%(3)	7%(1)	6%(1)	41%(9)	17%(1)	100%(2)	0%(0)
6-10 year	21%(20)	**25%(16)**	**14%(4)**	**0%(0)**	14%(3)	17%(1)	0%(0)	21%(3)	13%(2)	41%(9)	17%(1)	0%(0)	14%(1)
>10 years	39%(38)	**33%(21)**	**61%(17)**	**0%(0)**	29%(6)	33%(2)	0%(0)	64%(9)	81%(13)	14%(3)	33%(2)	0%(0)	43%(3)
*Total years’ experience: Palliative care (% within column total(n))*													
<1 year	30%(26)	30%(17)	32%(8)	20%(1)	35%(7)	33%(2)	0%(0)	21%(3)	23%(3)	19%(3)	50%(3)	50%(1)	57%(4)
1-5 years	46%(40)	53%(30)	24%(6)	80%(4)	60%(12)	33%(2)	100%(3)	43%(6)	8%(1)	75%(12)	50%(3)	50%(1)	0%(0)
6-10 year	14%(12)	12%(7)	20%(5)	0%(0)	5%(1)	0%(0)	0%(0)	21%(3)	39%(5)	6%(1)	0%(0)	0%(0)	29%(2)
>10 years	10%(9)	5%(3)	24%(6)	0%(0)	0%(0)	33%(2)	0%(0)	14%(2)	31%(4)	0%(0)	0%(0)	0%(0)	14%(1)
*Total years’ experience: Oncology (% within column total(n))*													
<1 year	20%(19)	**22%(14)**	**18%(5)**	**0%(0)**	29%(6)	33%(2)	0%(0)	14%(2)	6%(1)	5%(1)	33%(2)	0%(0)	83%(5)
1-5 years	44%(42)	**52%(33)**	**14%(4)**	**100%(5)**	67%(14)	33%(2)	100%(3)	7%(1)	0%(0)	82%(18)	33%(2)	100%(2)	0%(0)
6-10 year	12%(11)	**8%(5)**	**21%(6)**	**0%(0)**	5%(1)	0%(0)	0%(0)	14%(2)	31%(5)	5%(1)	17%(1)	0%(0)	17%(1)
>10 years	25%(24)	**18%(11)**	**46%(13)**	**0%(0)**	0%(0)	33%(2)	0%(0)	64%(9)	63%(10)	9%(2)	17%(1)	0%(0)	0%(0)
*Current practice area^ (% within column total(n))*													
Medical oncology	54%(52)	55%(35)	46%(13)	80%(4)	95%(20)	67%(4)	67%(2)	21%(3)	19%(3)	50%(11)	100%(6)	100%(2)	14%(1)
Surgical oncology	26%(25)	30%(19)	18%(5)	20%(1)	57%(12)	0%(0)	0%(0)	29%(4)	31%(5)	14%(3)	0%(0)	50%(1)	0%(0)
Radiation oncology	12%(12)	3%(2)	25%(7)	60%(3)	0%(0)	67%(4)	100%(3)	14%(2)	19%(3)	0%(0)	0%(0)	0%(0)	0%(0)
Palliative care	59%(57)	63%(40)	46%(13)	80%(4)	71%(15)	50%(3)	67%(2)	57%(8)	38%(6)	45%(10)	67%(4)	100%(2)	100%(7)
Pediatrics	2%(2)	2%(1)	4%(1)	0%(0)	0%(0)	0%(0)	0%(0)	7%(1)	6%(1)	0%(0)	0%(0)	0%(0)	0%(0)
General inpatient ward	6%(6)	8%(5)	4%(1)	0%(0)	24%(5)	0%(0)	0%(0)	0%(0)	0%(0)	0%(0)	17%(1)	0%(0)	0%(0)
General outpatient	1%(1)	2%(1)	0%(0)	0%(0)	0%(0)	0%(0)	0%(0)	7%(1)	0%(0)	0%(0)	0%(0)	0%(0)	0%(0)
Other	6%(6)	2%(1)	18%(5)	0%(0)	0%(0)	0%(0)	0%(0)	0%(0)	31%(5)	0%(0)	0%(0)	0%(0)	14%(1)
*Formal training in Palliative Care or Cancer Pain Management (% within column total(n))*													
No	51%(49)	**42%(27)**	**61%(17)**	**100%(5)**	33%(7)	67%(4)	100%(3)	43%(6)	44%(7)	**50%(11)**	**100%(6)**	**100%(2)**	43%(3)
Yes	49%(48)	**58%(37)**	**39%(11)**	**0%(0)**	67%(14)	33%(2)	0%(0)	57%(8)	56%(9)	**50%(11)**	**0%(0)**	**0%(0)**	57%(4)
*Types of training^ (Asked of those who said ‘yes’ to completing formal training) (% within column total(n))*													
Workshops (1-3 days)	51%(24)	46%(17)	70%(7)	-	43%(6)	0%(0)	-	38%(3)	78%(7)	46%(5)	-	-	75%(3)
Training courses (weeks-months)	60%(28)	68%(25)	30%(3)	-	64%(9)	100%(1)	-	88%(7)	22%(2)	82%(9)	-	-	0%(0)
Certificate programs (months-years)	9%(4)	8%(3)	10%(1)	-	0%(0)	0%(0)	-	13%(1)	11%(1)	9%(1)	-	-	25%(1)
Fellowships (months-years)	0%(0)	0%(0)	0%	-	0%(0)	0%(0)	-	0%(0)	0%(0)	0%(0)	-	-	0%(0)
Other	4%(2)	5%(2)	0%	-	0%(0)	0%(0)	-	0%(0)	0%(0)	0%(0)	-	-	50%(2)
How helpful in your clinical practice* (Asked of those who said ‘yes’ to completing formal training) (% within column total(n))													
Mean(n)	8.4(48)	**8.7(37)**	**7.5(11)**	-	**9.1(14)**	**7.0(2)**	-	8.4(8)	7.7(9)	8.2(11)	-	-	9.0(4)
*Time spent caring for patients with cancer (% within column total(n))*													
<10%	1%(1)	2%(1)	0%(0)	0%(0)	0%(0)	0%(0)	0%(0)	0%(0)	0%(0)	0%(0)	0%(0)	0%(0)	14%(1)
10-29%	1%(1)	2%(1)	0%(0)	0%(0)	0%(0)	0%(0)	0%(0)	0%(0)	0%(0)	5%(1)	0%(0)	0%(0)	0%(0)
30-49%	4%(4)	5%(3)	4%(1)	0%(0)	5%(1)	0%(0)	0%(0)	7%(1)	0%(0)	5%(1)	17%(1)	0%(0)	0%(0)
50-80%	43%(41)	42%(27)	48%(13)	20%(1)	29%(6)	40%(2)	0%(0)	21%(3)	50%(8)	77%(17)	50%(3)	50%(1)	14%(1)
>80%	51%(49)	50%(32)	48%(13)	80%(4)	67%(14)	60%(3)	100%(3)	71%(10)	50%(8)	14%(3)	33%(2)	50%(1)	71%(5)
*Highest completed level of education (% within column total(n))*													
Professional Certificate Level	25%(24)	**38%(24)**	**0%(0)**	**0%(0)**	**45%(9)**	**0%(0)**	**0%(0)**	**21%(3)**	**0%(0)**	**36%(8)**	**0%(0)**	**0%(0)**	57%(4)
Bachelors	41%(39)	**52%(33)**	**11%(3)**	**60%(3)**	**50%(10)**	**0%(0)**	**100%(3)**	**50%(7)**	**6%(1)**	**64%(14)**	**33%(2)**	**0%(0)**	29%(2)
Post-graduate	33%(32)	**10%(6)**	**86%(24)**	**40%(2)**	**5%(1)**	**100%6)**	**0%(0)**	**29%(4)**	**94%(15)**	**0%(0)**	**50%(3)**	**100%(2)**	14%(1)
Other	1%(1)	**0%(0)**	**4%(1)**	**0%(0)**	**0%(0)**	**0%(0)**	**0%(0)**	**0%(0)**	**0%(0)**	**0%(0)**	**17%(1)**	**0%(0)**	0%(0)

^a^ “Other" category comprised of 2 pharmacists and 3 trainees/students. A cell containing “ –“ indicates there were no responses from this role/category of provider on that question, while 0% means that 0% of this role/category of provider chose this answer.

* 0=not helpful, 10=very helpful

^ Respondents could select more than 1 response category. The column percentage is calculated based on the number of people who responded to the question; therefore, total percent can exceed 100. (n) = frequency of response for that item. Statistical significance was not tested on multiple response items.

^#^ Average mean score reported, based on scale of 0 (not helpful) to 10 (very helpful).

Bold numbers indicate the difference among roles/categories of provider on the question is statistically significant (α=0.05) either within the institution or between the overall nurse/physician group.

### Section 2: knowledge, beliefs and perceived barriers in managing cancer pain

#### Confidence and frequency of cancer pain management activities: Table 2

Participants were asked to rate their confidence (0, least confidence to 10, most confidence) in specific cancer pain management activities. For physicians and nurses (*n* = 92) the average mean confidence score for managing cancer pain, in general, was 7.8. Assessing the patient’s need for morphine, calculating breakthrough morphine doses, and adjusting/titrating morphine doses also all had high mean confidence scores (8.3; 8.4; 7.8, respectively).

**Table 2:** Comparison of mean confidence scores, by institution and by role, related to cancer pain management activity, 0 (no confidence) to 10 (very confident)

	Overall			Public General Hospital		Public Cancer Hospital		Private Cancer Hospital		Hospice
	100% (92)			29% (27)		33% (30)		30% (28)		8% (7)
Role	Total	Nurse	Physician	Nurse	Physician	Nurse	Physician	Nurse	Physician	Nurse
*%(n)*	100%(92)	70%(64)	30% (28)	78% (21)	22% (6)	47% (14)	53% (16)	79% (22)	21% (6)	100% (7)
*Cancer pain management activity*	Mean(n)	Mean(n)	Mean(n)	Mean(n)	Mean(n)	Mean(n)	Mean(n)	Mean(n)	Mean(n)	Mean(n)
Manage cancer pain in general	7.79(92)	7.77(64)	7.86(28)	8.24(21)	8.67(6)	7.50(14)	7.44(16)	7.45(22)	8.17(6)	7.86(7)
Assess patient’s need for morphine	8.25(92)	8.28(64)	8.18(28)	8.71(21)	8.83(6)	8.50(14)	7.75(16)	7.73(22)	8.67(6)	8.29(7)
Calculate breakthrough morphine doses	8.40(92)	8.59(64)	7.96(28)	8.57(21)	9.50(6)	**9.14(14)**	**7.00(16)**	8.32(22)	9.00(6)	8.43(7)
Adjust/titrate morphine doses	7.77(91)	7.73(63)	7.86(28)	7.95(20)	9.33(6)	**8.50(14)**	**6.94(16)**	**6.95(22)**	**8.83(6)**	8.00(7)
Prescribe morphine	8.23(31)	7.00(3)	8.36(28)	7.50(2)	9.33(6)	–	7.75(16)	–	9.00(6)	6.00(1)
Prescribe codeine	7.58(31)	7.33(3)	7.61(28)	8.00(2)	8.17(6)	–	7.13(16)	–	8.33(6)	6.00(1)
Prescribe tramadol	8.29(31)	7.67(3)	8.36(28)	8.50(2)	9.50(6)	–	7.56(16)	–	9.33(6)	6.00(1)
Prescribe fentanyl	6.90(31)	5.33(3)	7.07(28)	5.00(2)	7.67(6)	–	6.25(16)	–	8.67(6)	6.00(1)
Administer morphine	8.91(80)	9.02(64)	8.50(16)	**9.62(21)**	**10.00(3)**	8.93(14)	8.15(13)	8.77(22)	–	8.14(7)
Administer codeine	8.01(80)	8.08(64)	7.75(16)	**9.05(21)**	**10.00(3)**	8.36(14)	7.23(13)	6.95(22)	–	8.14(7)
Administer tramadol	8.61(80)	8.75(64)	8.06(16)	9.29(21)	10.00(3)	8.43(14)	7.62(13)	8.59(22)	–	8.29(7)
Administer fentanyl	7.41(78)	7.48(62)	7.13(16)	7.19(21)	9.33(3)	8.00(13)	6.62(13)	7.90(21)	–	6.14(7)
Dispense morphine	8.69(13)	8.45(11)	10.00(2)	8.75(4)	10.00(2)	–	–	–	–	8.29(7)
Dispense codeine	8.62(13)	8.36(11)	10.00(2)	8.75(4)	10.00(2)	–	–	–	–	8.14(7)
Dispense tramadol	8.69(13)	8.45(11)	10.00(2)	8.75(4)	10.00(2)	–	–	–	–	8.29(7)
Dispense fentanyl	6.77(13)	6.36(11)	9.00(2)	6.00(4)	9.00(2)	–	–	–	–	6.57(7)

Instructions provided in questionnaire: prescribe means to write an order; administer means to give the medication to a patient/family caregiver; dispense means to distribute the medication from a pharmacy

“– “indicates no responses from this role/category of provider

Bold numbers indicate the mean difference between nurses and physicians on the question is statistically significant (α = 0.05)

**Fig. 1:**
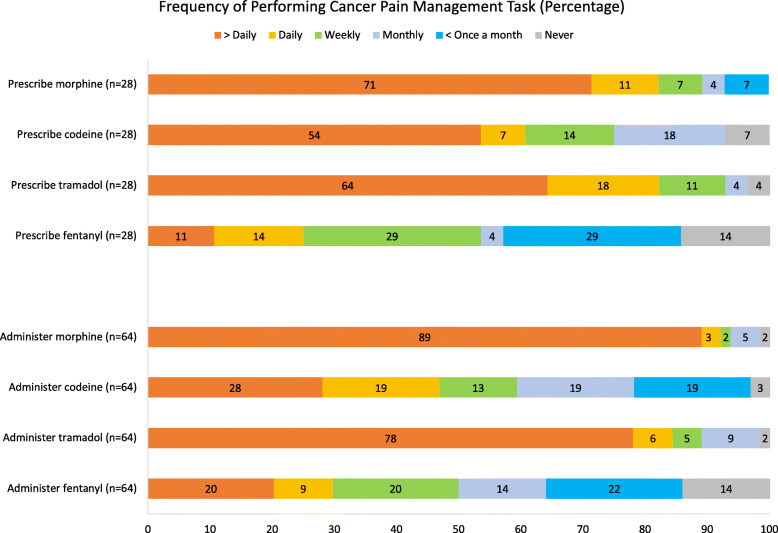
Frequency of performing cancer pain management task per opioid, for physicians (prescribing) and nurses (administering)

Statistically significant differences were found between nurses and physicians related to administering morphine and codeine at the public general hospital; calculating breakthrough morphine doses and adjusting/titrating morphine doses at the public cancer hospital; and adjusting/titrating morphine doses at the private cancer hospital. Frequency of prescribing (for physicians) and administering (for nurses) was also assessed per common opioid (Fig. 1). The most frequently prescribed opioid (> daily) by physicians was morphine (71%), followed by tramadol (64%), codeine (54%) and fentanyl (11%). The most frequently (> daily) administered opioid by nurses was morphine (89%), followed by tramadol (78%), codeine (28%), and fentanyl (20%).

#### Barriers to cancer pain management: Table 3

Potential barriers to cancer pain management were assessed in the categories of personal, institutional, and country/cultural factors (0, no impact to 10, significant impact). By overall category, the highest rated barriers were country/cultural (6.4, *n* = 92), followed by institutional (3.7, *n* = 91). The lowest category of barriers involved personal factors (3.0; *n* = 91).

**Table 3:** Comparison of mean impact scores of perceived barriers to cancer pain management, by institution and by role, (0, no impact; 10, significant impact)

	Overall			Public General Hospital		Public Cancer Hospital		Private Cancer Hospital		Hospice
	100% (92)			29% (27)		33% (30)		30% (28)		8% (7)
Role	Total	Nurse	Physician	Nurse	Physician	Nurse	Physician	Nurse	Physician	Nurse
*%(n)*	100%(92)	70%(64)	30% (28)	78% (21)	22% (6)	47% (14)	53% (16)	79% (22)	21% (6)	100% (7)
*Barriers to cancer pain management*	*Mean(n)*	*Mean(n)*	*Mean(n)*	*Mean(n)*	*Mean(n)*	*Mean(n)*	*Mean(n)*	*Mean(n)*	*Mean(n)*	*Mean(n)*
***Personal Factors (overall)***	3.04(91)	2.97(64)	3.22(27)	5.08(21)	2.2(5)	3.08(14)	4.39(16)	0.77(22)	0.95(6)	3.30(7)
I am not sure of the best action to take to manage cancer pain	3.51(91)	3.36(64)	3.85(27)	6.00(21)	2.00(5)	**2.71(14)**	**5.81(16)**	**1.18(22)**	**0.17(6)**	3.57(7)
I do not have enough time	2.97(91)	2.78(64)	3.41(27)	5.90(21)	2.40(5)	2.79(14)	4.94(16)	0.36(22)	0.17(6)	1.00(7)
I have to care for too many patients	4.19(91)	3.94(64)	4.78(27)	**6.86(21)**	**2.20(5)**	4.36(14)	6.31(16)	0.82(22)	2.83(6)	4.14(7)
I cannot obtain the proper order for pain management from the physician	3.21(85)	3.21(63)	3.23(22)	5.05(21)	2.25(4)	3.64(14)	4.92(12)	1.14(22)	0.50(6)	3.33(6)
I worry patients will get addicted to pain medication	2.30(91)	2.14(63)	2.64(28)	3.48(21)	3.33(6)	2.38(13)	3.25(16)	0.45(22)	0.33(6)	3.00(7)
I worry patients will suffer side effects from pain medication	3.12(91)	3.28(64)	2.74(27)	4.52(21)	2.40(5)	3.71(14)	3.25(16)	1.05(22)	1.67(6)	5.71(7)
I worry I will get in trouble for giving patients pain medication	2.15(91)	2.06(64)	2.37(27)	3.76(21)	2.20(5)	1.86(14)	2.94(16)	0.41(22)	1.00(6)	2.57(7)
***Institutional Factors (overall)***	3.71(91)	3.46(63)	4.26(28)	6.13(20)	5.40(6)	3.65(14)	5.20(16)	0.85(22)	0.65(6)	3.65(7)
The proper therapies of medications are not always available at my institution	3.21(92)	2.89(64)	3.93(28)	5.05(21)	3.50(6)	3.79(14)	5.44(16)	0.41(22)	0.33(6)	2.43(7)
The proper therapies or medications are too expensive for my patients to afford	3.28(92)	2.97(64)	4.00(28)	5.24(21)	5.17(6)	2.79(14)	4.38(16)	0.59(22)	1.83(6)	4.00(7)
Lack of palliative care or pain management training offered	3.99(91)	4.03(63)	3.89(28)	6.25(20)	5.00(6)	4.79(14)	4.94(16)	**0.95(22)**	**0.00(6)**	5.86(7)
Nurses and doctors do not communicate well	3.09(92)	2.73(64)	3.89(28)	4.48(21)	4.83(6)	4.57(14)	5.00(16)	**0.36(22)**	**0.00(6)**	1.29(7)
Volume of patients seen at the institution is too high	3.90(90)	3.50(62)	4.79(28)	5.05(20)	4.67(6)	5.07(14)	6.38(16)	1.23(22)	0.67(6)	3.00(6)
No/too few trained palliative care or pain management providers on staff	3.86(92)	3.63(64)	4.39(28)	5.24(21)	5.17(6)	3.50(14)	5.38(16)	1.91(22)	1.00(6)	4.43(7)
No separate beds or wards for palliative care	3.98(90)	3.60(62)	4.82(28)	8.11(19)	7.83(6)	**2.43(14)**	**5.13(16)**	0.64(22)	1.00(6)	3.00(7)
No palliative care outpatient department	4.22(91)	4.14(63)	4.39(28)	9.00(20)	7.00(6)	**2.29(14)**	**4.94(16)**	0.68(22)	0.33(6)	4.86(7)
***Country / Cultural Factors (overall)***	6.36(92)	6.55(64)	5.94(28)	**7.37(21)**	**5.00(6)**	5.93(14)	6.16(16)	6.24(22)	6.29(6)	6.27(7)
Patient or patient family worries the patient will get addicted to pain medication	6.00(92)	**6.38(64)**	**5.14(28)**	**7.67(21)**	**3.17(6)**	6.21(14)	5.38(16)	5.45(22)	6.50(6)	5.71(7)
Patient believes they should bear pain without complaint	5.11(92)	**5.53(64)**	**4.14(28)**	**5.86(21)**	**2.83(6)**	6.00(14)	4.69(16)	4.86(22)	4.00(6)	5.71(7)
Patient believes pain is deserved for wrongs committed in past lives	4.35(92)	4.58(64)	3.82(28)	5.05(21)	2.67(6)	5.43(14)	4.81(16)	3.64(22)	2.33(6)	4.43(7)
Patient family does not want the patient to know their diagnosis or prognosis	6.52(92)	6.75(64)	6.00(28)	7.05(21)	4.67(6)	6.79(14)	6.50(16)	6.82(22)	6.00(6)	5.57(7)
Patients live in rural areas and have trouble accessing healthcare services	7.33(92)	7.19(64)	7.64(28)	8.43(21)	7.50(6)	5.36(14)	7.38(16)	7.23(22)	8.50(6)	7.00(7)
Stigma and fear of cancer causes patients to delay treatment until the disease is very advanced and pain is very difficult	7.27(92)	7.38(64)	7.04(28)	8.29(21)	6.33(6)	6.57(14)	6.94(16)	7.27(22)	8.00(6)	6.57(7)
Nursing and medical school curriculums in Nepal do not contain enough content about palliative care and pain management	7.96(92)	8.03(64)	7.79(28)	9.29(21)	7.83(6)	5.14(14)	7.44(16)	8.41(22)	8.67(6)	8.86(7)

Bold numbers indicate the mean difference between nurses and physicians is statistically significant (α = 0.05)

The highest rated country/cultural barrier, overall (mean 8.0, *n* = 92) and by nurses (mean 8.0, *n* = 64) and physicians (mean 7.8, *n* = 28) was ‘nursing and medical school curriculums in Nepal do not contain enough content about palliative care and pain management.’ Other top ranked system-level barriers included ‘patients in rural areas have trouble accessing healthcare services’ (overall mean, 7.3, *n* = 92) and ‘stigma and fear of cancer cause delayed diagnosis/treatment’ (overall mean 7.3, *n* = 92). The lowest ranked country/cultural barrier overall (mean 4.4, *n* = 92) and by nurses (mean 4.6, *n* = 64) and physicians (mean 3.8, *n* = 28) was ‘patient believes pain is deserved for wrongs committed in past lives.’ Statistically significant differences were found between nurses and physicians overall, and within the public general hospital, related to the barriers ‘patient or patient’s family worries the patient will get addicted to pain medication’ and ‘patient believes they should bear pain without complaint,’ with nurses rating these barriers with greater impact.

The highest rated institutional barrier, overall (mean 4.2, *n* = 91) and by nurses (mean 4.1, *n* = 63) was ‘no palliative care outpatient department.’ For physicians, the highest rated institutional barrier was ‘no separate beds or wards for palliative care’ (mean 4.8; *n* = 28). The lowest ranked institutional barrier, overall (mean 3.1, *n* = 92) and by nurses (mean 2.7, *n* = 64) and physicians (mean 3.9, *n* = 28) was ‘nurses and doctors do not communicate well;’ this barrier was significantly different within the private cancer hospital, with all physician respondents ranking this factor as having no impact (mean 0.0, *n* = 6).

The highest rated personal barrier was ‘I have to care for too many patients,’ (overall 4.2, *n* = 91; nurses 3.9, *n* = 64; physicians 4.8, *n* = 27). This barrier was statistically significant between nurses (mean 6.9; *n* = 21) and physicians (mean 2.2; *n* = 5) at the public general hospital. The lowest rated personal barriers included “I worry I will get in trouble for giving patients pain medication,” (overall 2.2, *n* = 91; nurses 2.1, *n* = 64; physicians 2.4, *n* = 27) and “I worry patients will get addicted to pain medication,” (overall 2.3, *n* = 91; nurses 2.1, *n* = 64; physicians 2.6, *n* = 28). Ratings for “I am not sure of the best action to take to manage cancer pain” varied significantly between nurses and physicians at both the public cancer hospital and the private cancer hospital. However, the physicians at the public cancer hospital rated it significantly higher than nurses at their institution (physicians 5.8, *n* = 16; nurses 2.7, *n* = 14), whereas physicians at the private cancer hospital rated the barrier significantly lower than nurses (physicians 0.2, *n* = 6; nurses 1.2, *n* = 22).

#### Knowledge and beliefs related to cancer pain management: Table 4

On a scale of 0, strongly disagree to 10, strongly agree, 95% of respondents reported an agreement rating of 8 or higher with the statement ‘it is my role/my job to manage cancer pain.’ While this item had a high overall rating (overall mean 9.3, *n* = 92), there was a statistically significant difference between nurses (mean 9.5, *n* = 64) and physicians (mean = 8.9, *n* = 28). 92% of respondents also agreed or strongly agreed (8 or higher) that morphine is a beneficial and helpful medication (overall mean 8.9, *n* = 83; nurses mean 8.9, *n* = 56; physicians mean 9.0, *n* = 27). 82% of respondents disagreed or strongly disagreed (2 or lower) that morphine is only appropriate for dying patients (overall mean 1.0, *n* = 91; nurses mean 1.1, *n* = 63; physicians mean 0.9, *n* = 28). 45% of respondents also disagreed or strongly disagreed (2 or lower) that ‘regular morphine use will cause physical dependence,’ (overall mean 3.6, *n* = 90; nurses mean 3.7, *n* = 62; physicians mean 3.4, *n* = 28). 67% of respondents disagreed or strongly disagreed (2 or lower) that ‘regular morphine use will cause addiction.’ Lastly, 66% of respondents disagreed or strongly disagreed (2 or lower) that ‘morphine commonly causes respiratory depression’ (overall mean 2.4, *n* = 88; nurses mean 2.8, *n* = 60; physician mean 1.7, *n* = 28) and this difference was statistically significant between nurses and physicians at the public cancer hospital (nurse mean 4.6, *n* = 14; physician mean 2.1, *n* = 16) and the private cancer hospital (nurse mean 2.6, *n* = 21; physician mean 1.0, *n* = 6).

**Table 4:** Knowledge and beliefs related to cancer pain management, by institution and by role

	Overall			Public General Hospital		Public Cancer Hospital		Private Cancer Hospital		Hospice
	100% (92)			29% (27)		33% (30)		30% (28)		8% (7)
Role	Total	Nurse	Physician	Nurse	Physician	Nurse	Physician	Nurse	Physician	Nurse
*%(n)*	100%(92)	70%(64)	30% (28)	78% (21)	22% (6)	47% (14)	53% (16)	79% (22)	21% (6)	100% (7)
	*Mean(n)*	*Mean(n)*	*Mean(n)*	*Mean(n)*	*Mean(n)*	*Mean(n)*	*Mean(n)*	*Mean(n)*	*Mean(n)*	*Mean(n)*
Opioid Knowledge / Beliefs*										
It is my role/my job to manage cancer pain	9.29(92)	**9.45(64)**	**8.93(28)**	9.71(21)	9.50(6)	9.29(14)	8.56(16)	9.41(22)	9.33(6)	9.14(7)
Regular morphine use will cause addiction	2.18(91)	1.94(63)	2.71(28)	2.14(21)	3.83(6)	3.62(13)	2.94(16)	0.73(22)	1.00(6)	2.00(7)
Regular morphine use will cause physical dependence	3.64(90)	3.74(62)	3.43(28)	7.32(19)	6(6)	3.79(14)	3.25(16)	0.68(22)	1.33(6)	3.57(7)
Morphine is a beneficial and helpful medication	8.93(83)	8.89(56)	9.00(27)	9.26(19)	9.33(6)	8.67(12)	8.69(16)	8.56(18)	9.6(5)	9.14(7)
Morphine is only appropriate for dying patients	1.03(91)	1.11(63)	0.86(28)	1.15(20)	0.50(6)	1.93(14)	1.25(16)	0.77(22)	0.17(6)	0.43(7)
Morphine commonly causes respiratory depression	2.43(88)	2.77(60)	1.71(28)	1.61(18)	1.33(6)	**4.64(14)**	**2.13(16)**	**2.57(21)**	**1.00(6)**	2.57(7)
*% within column total (n)*										
Personal feelings about cancer pain**										
Cancer pain is unavoidable and cannot be controlled	0%(0)	0%(0)	0%(0)	0%(0)	0%(0)	0%(0)	0%(0)	0%(0)	0%(0)	0%(0)
Cancer pain can be difficult but usually can be controlled	95%(87)	97%(62)	89% (25)	95% (20)	83% (5)	100% (14)	88% (14)	95% (21)	100% (6)	100% (7)
Cancer pain is not a problem for my patients	4% (4)	3% (2)	7% (2)	5% (1)	17% (1)	0%(0)	6% (1)	5% (1)	0%(0)	0%(0)
Other	1% (1)	0%(0)	4% (1)	0%(0)	0%(0)	0%(0)	6% (1)	0%(0)	0%(0)	0%(0)

Bold numbers indicate the difference between nurses and physicians on the question is statistically significant (α = 0.05)

*Comparison of mean agreement scores; 0 = strongly disagree, 10 = strongly agree

**Comparison of percentages and frequencies; for this question, respondents were asked to endorse/select only one response

The majority of respondents (95%, *n* = 87) endorsed the statement ‘cancer pain can be difficult but usually can be controlled.’ No respondents endorsed the statement “cancer pain is unavoidable and cannot be controlled.’

#### Reported opioid availability: Table 5

The entire sample (*n* = 97) is included in Table 5 to capture the insights of pharmacists. Overall, 94–100% of respondents reported that all morphine formulations are regularly available at their institution. Shortages of morphine, codeine, and tramadol were rarely reported within the past 6 months at any institution; fentanyl shortages were more common.

**Table 5:** Reported opioid availability by institution, total sample

	Overall	Public General Hospital	Public Cancer Hospital	Private Cancer Hospital	Hospice Nepal
	100% (97)	31% (30)	31% (30)	31% (30)	7% (7)
*% within column total (n)*					
*Morphine formulations regularly available*^*a*^					
10 mg immediate release	94%(90)	100% (30)	80% (24)	100% (30)	100% (6)
10 mg prolonged release	97%(93)	97% (29)	93% (28)	100% (30)	100% (6)
30 mg prolonged release	95%(91)	97% (29)	87% (26)	100% (30)	100% (6)
Morphine syrup	100%(96)	100% (30)	100% (30)	100% (30)	100% (6)
Morphine injectable	95%(91)	93% (28)	90% (27)	100% (30)	100% (6)
None of these are regularly available	0%(0)	0%(0)	0%(0)	0%(0)	0%(0)
Unsure	0%(0)	0%(0)	0%(0)	0%(0)	0%(0)
*Shortages within past 6 months*					
*Morphine*					
Yes	6% (6)	10% (3)	7% (2)	0%(0)	14% (1)
No	91%(88)	87% (26)	87% (26)	100% (30)	86% (6)
Unsure	3% (3)	3% (1)	7% (2)	0%(0)	0%(0)
*Average duration of morphine shortage (Asked of those reporting morphine shortage)*					
A few days	50% (7)	20% (1)	25% (1)	–	100% (5)
A few weeks	7% (1)	20% (1)	0%(0)	–	0%(0)
A few months	7% (1)	20% (1)	0%(0)	–	0%(0)
More than a few months	0%(0)	0%(0)	0%(0)	–	0%(0)
Unsure how long lasted	36% (5)	40% (2)	75% (3)	–	0%(0)
*Codeine*					
Yes	5% (5)	7% (2)	7% (2)	0%(0)	14% (1)
No	93%(89)	87% (26)	93% (28)	100% (29)	86% (6)
Unsure	2% (2)	7% (2)	0%(0)	0%(0)	0%(0)
*Average duration of codeine shortage (Asked of those reporting codeine shortage)*					
A few days	15% (2)	33% (2)	0%(0)	–	0%(0)
A few weeks	8% (1)	0%(0)	50% (1)	–	0%(0)
A few months	8% (1)	17% (1)	0%(0)	–	0%(0)
More than a few months	0%(0)	0%(0)	0%(0)	–	0%(0)
Unsure how long lasted	69% (9)	50% (3)	50% (1)	–	100% (5)
*Tramadol*					
Yes	4% (4)	3% (1)	7% (2)	0%(0)	14% (1)
No	95%(92)	93% (28)	93% (28)	100% (30)	86% (6)
Unsure	1% (1)	3% (1)	0%(0)	0% (1)	0%(0)
*Average duration of tramadol shortage (Asked of those reporting tramadol shortage)*					
A few days	9% (1)	25% (1)	0%(0)	–	0%(0)
A few weeks	9% (1)	0%(0)	50% (1)	–	0%(0)
A few months	9% (1)	25% (1)	0%(0)	–	0%(0)
More than a few months	0%(0)	0%(0)	0%(0)	–	0%(0)
Unsure how long lasted	73% (8)	50% (2)	50% (1)	–	100% (5)
*Fentanyl*					
Yes	27% (26)	**7%(2)**	**23%(7)**	**50%(15)**	**33%(2)**
No	47% (45)	**53%(16)**	**40%(12)**	**47%(14)**	**50%(3)**
Unsure	26% (25)	**40%(12)**	**37%(11)**	**3%(1)**	**17%(1)**
*Average duration of fentanyl shortage (Asked of those reporting fentanyl shortage)*					
A few days	32% (13)	**13%(1)**	**33%(4)**	**25%(4)**	**80%(4)**
A few weeks	7% (3)	**0%(0)**	**8%(1)**	**6%(1)**	**20%(1)**
A few months	24% (10)	**0%(0)**	**0%(0)**	**63%(10)**	**0%(0)**
More than a few months	0%(0)	**0%(0)**	**0%(0)**	**0%(0)**	**0%(0)**
Unsure how long lasted	37% (15)	**88%(7)**	**58%(7)**	**6%(1)**	**0%(0)**

^*a*^ Respondents could select more than 1 response category. The column percentage is calculated based on the number of people who responded to the question; therefore, total percent can exceed 100. (n) = frequency of response for that item. Statistical significance was not tested on multiple response items

“– “indicates there were no responses from this hospital on that question, while 0% means that 0% of respondents from this hospital chose this answer

Bold numbers indicate the difference among hospitals on the question are statistically significant (α = 0.05)

Specifically, the most frequently reported regularly available morphine formulation overall was morphine syrup, which was available at every institution (100%; *n* = 96), followed by morphine 10 mg prolonged release (97%, *n* = 93), morphine 30 mg prolonged release and morphine injectable (both 95%, *n* = 91), and then morphine 10 mg immediate release (94%, *n* = 90). No respondent selected the option ‘none of these morphine formulations are regularly available at my institution’ or ‘unsure.’

The majority of respondents (91%, *n* = 88) reported no morphine shortages within the past 6 months at their institution; a small number of respondents indicated a morphine shortage had occurred at their institution in the past 6 months (6%, *n* = 6) or they were unsure (3%, *n* = 3). Overall, respondents indicated that if morphine shortages had occurred, they generally lasted a few days (50%, *n* = 7) or they were unsure of duration (36%, *n* = 5). Shortages within the past 6 months of codeine and tramadol were also infrequently reported (93%, *n* = 89 stating no shortage of codeine; 95%, *n* = 92 stating no shortage of tramadol), and if they did occur, respondents were more unsure of duration (unsure duration of codeine shortage, 69%, *n* = 9; unsure duration of tramadol shortage, 73%, *n* = 8). Overall and between institutions there was the most variance regarding fentanyl, with reported shortages and duration of fentanyl shortages statistically different among the study sites (occurring more frequently and of longer duration at the private cancer hospital) and more respondents overall reporting fentanyl shortages (27%, *n* = 26) or being unsure if fentanyl shortages occurred (26%, *n* = 25). Of note, the survey did not specify formulation of fentanyl, e.g., transdermal patch versus parenteral.

#### Use of non-opioid and non-pharmacological therapies: Table 6

The most commonly reported non-opioid pharmacological treatments for cancer pain were non-steroidal anti-inflammatory drugs (NSAIDs, 96%, *n* = 88), paracetamol (95%, *n* = 87), and steroids (91%, *n* = 84). The most commonly reported non-pharmacological treatments for cancer pain were heat/cold packs (82%, *n* = 75); massage (79%, *n* = 73); and meditation/yoga (35%, *n* = 32).

**Table 6:** Reported use of non-opioid pharmacological and non-pharmacological therapies to manage cancer pain, by institution, by role

	Overall			Public General Hospital		Public Cancer Hospital		Private Cancer Hospital		Hospice
	100% (92)			29%(27)		33%(30)		30%(28)		8%(7)
Role *%(n) within row total*	Total	Nurse	Physician	Nurse	Physician	Nurse	Physician	Nurse	Physician	Nurse
	100%(92)	70%(64)	30%(28)	78%(21)	22%(6)	47%(14)	53%(16)	79%(22)	21%(6)	100%(7)
Non-opioid pharmacological therapies used to manage cancer pain* % within column total (n)										
NSAIDs**	96%(88)	94%(60)	100%(28)	90%(19)	100%(6)	100%(14)	100%(16)	96%(21)	100%(6)	86%(6)
Steroids	91%(84)	92%(59)	89%(25)	90%(19)	100%(6)	93%(13)	81%(23)	91%(20)	100%(6)	100%( 7)
Paracetamol	95%(87)	92%(59)	100%(28)	95%(20)	100%(6)	86%(12)	100%(16)	96%(21)	100%(6)	86%(6)
Amitriptyline	90%(83)	91%(58)	89%(25)	86%(18)	83%(5)	93%(13)	88%(14)	91%(20)	100%(6)	100%(7)
Gabapentin	83%(76)	81%(52)	86%(24)	67%(14)	83%(5)	71%(10)	81%(13)	96%(21)	100%(6)	100%(7)
Pre-gabalin	88%(81)	89%(57)	86%(24)	95%(20)	100%(6)	64%(9)	75%(12)	96%(21)	100%(6)	100%(7)
Other^	18%(17)	15%(10)	25%(7)	0%(0)	33%(2)	7%(1)	25%(4)	9%(2)	17%(1)	100%(7)
I do not use any of these	1%(1)	0%(0)	4%(1)	0%(0)	0%(0)	0%(0)	6%(1)	0%(0)	0%(0)	0%(0)
Non-pharmacological therapies used to manage cancer pain* % within column total (n)										
Heat/cold packs	82%(75)	92%(59)	57%(16)	76%(16)	33%(2)	100%(14)	50%(8)	100%(22)	100%(6)	100%(7)
Massage	79%(73)	88%(56)	61%(17)	67%(14)	50%(3)	100%(14)	50%(8)	96%(21)	100%(6)	100%(7)
Acupuncture/acupressure	27%(25)	28%(18)	25%(7)	19%(4)	0%(0)	79%(11)	44%(7)	9%(2)	0%(0)	14%(1)
Meditation/yoga	35%(32)	38%(24)	29%(8)	29%(6)	33%(2)	86%(12)	38%(6)	5%(1)	0%(0)	71%(5)
TENS***	17%(16)	17%(11)	18%(5)	19%(4)	17%(1)	43%(6)	25%(4)	0%(0)	0%(0)	14%(1)
Other^^	21%(19)	22%(14)	18%(5)	38%(8)	33%(2)	7%(1)	19%(3)	0%(0)	0%(0)	71%(5)
I do not use any of these	8%(7)	2%(1)	21%(6)	5%(1)	33%(2)	0%(0)	25%(4)	0%(0)	0%(0)	0%(0)

* Respondents could select more than 1 response category. The column percentage is calculated based on the number of people who responded to the question; therefore, total percent can exceed 100. (n) = frequency of response for that item. Statistical significance was not tested on multiple response items.

**NSAIDs = non-steroidal anti-inflammatory drugs

***TENS = transcutaneous electrical nerve stimulation

^Including: e.g., nerve block, radiation therapy, ketamine, capsaicin, clonazepam, diclofenac, duloxetine, zoledronate.

^^Including: Physiotherapy, music, emotional support (i.e. communication, counseling, prayer), diversional therapy (i.e. television, reading, play, drawing).

### Section 3: awareness and use of the NAPCare PMG: Table 7

96% of nurses and physicians (*n* = 88) had heard of the NAPCare PMG; of those, 97% (*n* = 85) had read at least some, or all, of the guidelines. There were statistically significant differences between nurses and physicians related to ‘helpfulness of the guidelines in your clinical practice’ (nurses, 8.90, *n* = 53; physicians, 8.16, *n* = 25) and regarding frequency of use of the NAPCare guidelines in clinical practice, with nurses more likely to use the guidelines multiple times a day (84%, *n* = 53) compared to physicians (56%; *n* = 14). 37% (*n* = 32) of respondents indicated that they use other PMG guidelines to help manage cancer pain, usually the World Health Organization (WHO) guidelines (*n* = 23).

**Table 7:** Awareness and use of NAPCare Pain Management Guidelines (PMG) by institution and role

	Overall			Public General Hospital		Public Cancer Hospital		Private Cancer Hospital		Hospice
	100% (92)			29% (27)		33% (30)		30% (28)		8% (7)
Roles *% within row total*	Total	Nurse	Physician	Nurse	Physician	Nurse	Physician	Nurse	Physician	Nurse
	100%(92)	70%(64)	30% (28)	78% (21)	22% (6)	47% (14)	53% (16)	79% (22)	21% (6)	100% (7)
*Have heard of the NAPCare PMG % within column total (n)*										
Yes	96% (88)	98% (63)	89% (25)	95% (20)	100% (6)	100% (14)	81% (13)	100% (22)	100% (6)	100% (7)
No	4% (4)	2% (1)	11% (3)	5% (1)	0% (0)	0% (0)	19% (3)	0% (0)	0% (0)	0% (0)
*Source of Information on NAPCare PMG^ (Asked of those who heard about PMG) % within column total (n)*										
Professional colleague	65% (57)	65% (41)	64% (16)	50% (10)	67% (4)	57% (8)	46% (6)	86% (19)	100% (6)	57% (4)
Saw the booklet	76% (67)	79% (50)	68% (17)	80% (16)	83% (5)	80% (16)	54% (7)	100% (22)	83% (5)	43% (3)
Palliative care training	43% (38)	54% (34)	16% (4)	60% (12)	0%(0)	60% (12)	31% (4)	55% (12)	0%(0)	43% (3)
Other	3% (3)	2% (1)	8% (2)	0% (0)	17% (1)	0% (0)	8% (1)	0% (0)	0% (0)	14% (1)
*Have read NAPCare PMG (Asked of those who heard about PMG) % within column total (n)*										
Yes, all of it*	73% (64)	75% (47)	68% (17)	70% (14)	67% (4)	43% (6)	54% (7)	100% (22)	100% (6)	71% (5)
Yes, some of it*	24% (21)	22% (14)	28% (7)	30% (6)	33% (2)	43% (6)	39% (5)	0% (0)	0% (0)	29% (2)
No	3% (3)	3% (2)	4% (1)	0% (0)	0% (0)	14% (2)	8% (1)	0% (0)	0% (0)	0% (0)
*Use of NAPCare PMG in clinical practice (Asked of those who heard about PMG) % within column total (n)*										
Never	1% (1)	**0%(0)**	**4% (1)**	0% (0)	0% (0)	0% (0)	8% (1)	0%(0)	0% (0)	0% (0)
< Once a month	6% (5)	**3% (2)**	**12% (3)**	0% (0)	17% (1)	14% (2)	15% (2)	0% (0)	0% (0)	0% (1)
Once a month	2% (2)	**0%**	**8% (2)**	0% (0)	0% (0)	0% (0)	15% (2)	0% (0)	0% (0)	0% (1)
Once a week	7% (6)	**3% (2)**	**16% (4)**	5% (1)	33% (2)	0% (0)	15% (2)	0% (0)	0% (0)	14% (1)
Once a day	8% (7)	**10% (6)**	**4% (1)**	20% (4)	17% (1)	7% (1)	0% (0)	5% (1)	0% (0)	0% (1)
Multiple times per day	76% (67)	**84% (53)**	**56% (14)**	74% (15)	33% (2)	79% (11)	46% (6)	95% (21)	100% (6)	79% (11)
*How helpful in your clinical practice^^ (Asked of those who heard about PMG) % within column total (n)*										
Mean(n)	8.68(88)	**8.90(63)**	**8.16(25)**	9.35(20)	8.50(6)	8.21(14)	7.85(13)	9.00(22)	8.50(6)	8.57(7)
*Use of other PMG guidelines % within column total (n)*										
Yes	37% (32)	34% (21)	44% (11)	47% (9)	50% (3)	43% (6)	54% (7)	0% (0)	167% (1)	86% (6)
No	63% (55)	66% (41)	56% (14)	53% (10)	50% (3)	57% (8)	46% (6)	100% (22)	83% (5)	14% (1)
*Types of other PMG guidelines (Asked of those who reported use of other PMG) % within column total (n)*										
Use WHO Guidelines	72% (23)	95% (19)	44% (4)	100% (8)	33% (1)	100% (6)	60% (3)	–	–	83% (5)

“– “indicates there were no responses from this role/category of provider on that question, while 0% means that 0% of this role/category of provider chose this answer

^ Respondents could select more than 1 response category. The column percentage is calculated based on the number of people who responded to the question; therefore, total percent can exceed 100. (n) = frequency of response for that item. Statistical significance was not tested on multiple response items. ^^^^ Average mean score reported, based on scale of 0 (not helpful) to 10 (very helpful)

Bold numbers indicate the difference among roles/categories of provider on the question is statistically significant (α = 0.05) within the institution or between overall role

### Section 4: smart phone usage, barriers and desired feature of the mobile app: Table 8

All but two participants (98% of nurses and physicians; *n* = 90) reported having access to a smart phone; of-those, all respondents (*n* = 89) reported having daily access to a smart phone, and most (82%; *n* = 74) use an Android phone. 46% of physicians (*n* = 13) reported “I encounter no barriers in using a smart phone” compared to 3% (*n* = 2) of nurses. The most frequent barriers to smart phone use reported by physicians were cost of data access (*n* = 11; 39%) and concern about using smart phones in the presence of patients (*n* = 5; 18%). Nurses reported more barriers to smart phone use in general, the top three being cost of data access (*n* = 45; 70%); not allowed to use mobile phone at work (*n* = 39; 61%); and concern about using smart phones in the presence of patients (*n* = 34; 53%).

**Table 8:** Smart Phone usage, barriers and app preferences, by institution and role

	Overall			Public General		Public Cancer		Private Cancer		Hospice
	100% (92)			29% (27)		33% (30)		30% (28)		8% (7)
Roles *% within row total(n)*	Total	Nurse	Physician	Nurse	Physician	Nurse	Physician	Nurse	Physician	Nurse
	100%(92)	70%(64)	30% (28)	78% (21)	22% (6)	47% (14)	53% (16)	79% (22)	21% (6)	100% (7)
*Access to Smart Phone % within column total (n)*										
Yes	98%(90)	97%(62)	100% (28)	100% (21)	100% (6)	86% (12)	100% (16)	100% (22)	100% (6)	100% (7)
No	2% (2)	3% (2)	0%(0)	0%(0)	0%(0)	14% (2)	0%(0)	0%(0)	0%(0)	0%(0)
*Type of Smart Phone Used* *(Asked of those with access to a smart phone) % within column total (n)*										
Android	82%(74)	87% (54)	71% (20)	81% (17)	83% (5)	92% (11)	63% (10)	96% (21)	83% (5)	71% (5)
IOS	17% (15)	11% (7)	29% (8)	19% (4)	17% (1)	8% (1)	38% (6)	5% (1)	17% (1)	14% (1)
Other	1% (1)	2% (1)	0%(0)	0%(0)	0%(0)	0%(0)	0%(0)	0%(0)	0%(0)	14% (1)
*Frequency of Smart Phone Use* *(Asked of those with access to a smart phone) % within column total (n)*										
Daily	100%(89)	100%(61)	100% (28)	100% (20)	100% (6)	100% (12)	100% (16)	100% (22)	100% (6)	100% (7)
Few times/week	0%(0)	0%(0)	0%(0)	0%(0)	0%(0)	0%(0)	0%(0)	0%(0)	0%(0)	0%(0)
Few times/month	0%(0)	0%(0)	0%(0)	0%(0)	0%(0)	0%(0)	0%(0)	0%(0)	0%(0)	0%(0)
Few times/year	0%(0)	0%(0)	0%(0)	0%(0)	0%(0)	0%(0)	0%(0)	0%(0)	0%(0)	0%(0)
Other	0%(0)	0%(0)	0%(0)	0%(0)	0%(0)	0%(0)	0%(0)	0%(0)	0%(0)	0%(0)
*Barriers to Smart Phone use^ % within column total (n)*										
Lack of mobile service at home	3% (3)	5% (3)	0%(0)	0%(0)	0%(0)	7% (1)	0%(0)	5% (1)	0%(0)	14% (1)
Lack of mobile service at work	13% (12)	19% (12)	0%(0)	5% (1)	0%(0)	36% (5)	0%(0)	23% (5)	0%(0)	14% (1)
Not allowed to use mobile phone at work	43% (40)	61% (39)	4% (1)	81% (17)	0%(0)	43% (6)	0%(0)	68% (15)	17% (1)	14% (1)
Cost of data access	61% (56)	70% (45)	39% (11)	52% (11)	17% (1)	79% (11)	44% (7)	91% (20)	50% (3)	43% (3)
Cost of Smart Phone	15% (14)	22% (14)	0%(0)	5% (1)	0%(0)	29% (4)	0%(0)	32% (7)	0%(0)	29% (2)
Concern about using in presence of patients	42% (39)	53% (34)	18% (5)	48% (10)	0%(0)	14% (2)	25% (4)	82% (18)	17% (1)	57% (4)
I am not interested in using a Smart Phone	1% (1)	2% (1)	0%(0)	0%(0)	0%(0)	7% (1)	0%(0)	0%(0)	0%(0)	0%(0)
I encounter no barriers in using a Smart Phone	16% (15)	3% (2)	46% (13)	0%(0)	67% (4)	14% (2)	44% (7)	0%(0)	33% (2)	0%(0)
Other	2% (2)	2% (1)	4% (1)	0%(0)	17% (1)	0%(0)	0%(0)	0%(0)	0%(0)	14% (1)
*How could an app help better manage cancer pain^ % within column total (n)*										
Assess cancer pain	73%(67)	73% (47)	71% (20)	81% (17)	83% (5)	50% (7)	69% (11)	82% (18)	67% (4)	71% (5)
Understand cancer pain physiology	75%(69)	84% (54)	54% (15)	95% (20)	33% (2)	57% (8)	44% (7)	86% (19)	100% (6)	100% (7)
Prescribe/give opioid medications	78%(72)	73% (47)	89% (25)	86% (18)	100% (6)	43% (6)	81% (13)	91% (20)	100% (6)	43% (3)
Prescribe/give non-opioid medications	73%(67)	69% (44)	82% (23)	76% (16)	67% (4)	36% (5)	81% (13)	91% (20)	100% (6)	43% (3)
Prescribe/give non-pharmacological treatments	63%(58)	67% (43)	54% (15)	67% (14)	50% (3)	57% (8)	44% (7)	86% (19)	83% (5)	29% (2)
Educate patients/family members	86%(79)	92%(59)	71% (20)	100% (21)	83% (5)	71% (10)	63% (10)	100% (22)	83% (5)	86% (6)
Review reports of patient symptoms	66%(61)	69% (44)	61% (17)	81% (17)	67% (4)	43% (6)	50% (8)	82% (18)	83% (5)	43% (3)
Share information with/learn from other Healthcare-Providers (HCPs)	82%(75)	84% (54)	75% (21)	91% (19)	83% (5)	64% (9)	69% (11)	91% (20)	83% (5)	86% (6)
Other	4% (4)	6% (4)	0%(0)	5% (1)	0%(0)	7% (1)	0%(0)	0%(0)	0%(0)	29% (2)
*How to know if app to manage cancer pain is making a positive difference^ % within column total (n)*										
Patients will be in less pain	76%(70)	78% (50)	71% (20)	76% (16)	100% (6)	64% (9)	56% (9)	91% (20)	83% (5)	71% (5)
Families will be less stressed	79%(73)	84% (54)	68% (19)	81% (17)	83% (5)	79% (11)	50% (8)	91% (20)	100% (6)	86% (6)
HCPs will use the app often	79%(73)	78% (50)	82% (23)	86% (18)	67% (4)	57% (8)	88% (14)	91% (20)	83% (5)	57% (4)
HCPs will feel more confident prescribing/giving pain therapies	88%(81)	88% (56)	89% (25)	95% (20)	67% (4)	50% (7)	94% (15)	100% (22)	100% (6)	100% (7)
Cancer pain management guidelines will be followed more consistently	90%(83)	89%(57)	93% (26)	100% (21)	83% (5)	50% (7)	94% (15)	100% (22)	100% (6)	100% (7)
Morphine will be more available to patients in need	57% (52)	58% (37)	54% (15)	71% (15)	33% (2)	29% (4)	63% (10)	73% (16)	50% (3)	29% (2)
Other	4% (4)	5% (3)	4% (1)	5% (1)	0%(0)	7% (1)	6% (1)	0%(0)	0%(0)	14% (1)
*Frequency of app use, clinical care^^*										
Mean(n)	6.38(92)	6.33(64)	6.50(28)	6.14(21)	6.50(6)	5.86(14)	5.94(16)	7.18(22)	8.00(6)	5.14(7)
*Frequency of app use, personal reasons^^*										
Mean(n)	7.65(92)	7.44(64)	8.14(28)	8.81(21)	7.83(6)	**5.50(14)**	**8.6(16)**	7.55(22)	8.67(6)	6.86(7)

^ Respondents could select more than 1 response category. The column percentage is calculated based on the number of people who responded to the question; therefore, total percent can exceed 100. (n) = frequency of response for that item. Statistical significance was not tested on multiple response items

^^^^ Average mean score reported, based on scale of 0 (never) to 10 (very often)

Bold numbers indicate the difference among roles/categories of provider on the question is statistically significant (α = 0.05) within the institution or between overall provider role

HCPs, Healthcare-Providers

When asked how a mobile app could help healthcare providers better manage cancer pain, nurses most frequently selected ‘educating patients and family members’ (92%; *n* = 59), followed by ‘sharing information with/learning from other healthcare providers’ and ‘understanding cancer pain physiology’ (both 84%; *n* = 54). Physicians most frequently selected ‘prescribing/giving opioid medications’ (89%; *n* = 25), followed by ‘prescribing/giving non-opioid medications’ (82%; *n* = 23), and ‘sharing information with/learning from other healthcare providers’ (75%; *n* = 21). Both nurses and physicians selected the option ‘prescribing/giving non-pharmacological treatments’ as the least important way a mobile app could help them manage cancer pain (67%; *n* = 43; 54%, *n* = 15, respectively). Overall, nurses and physicians most frequently selected the option ‘cancer pain management guidelines will be followed more consistently’ (90%; *n* = 83) and ‘healthcare providers will feel more confident prescribing/giving pain therapies’ (88%; *n* = 81) as the top indictors to evaluate app effectiveness; ‘morphine will be more available to patients in need’ received the fewest responses as a way to evaluate the app’s effectiveness (57%; *n* = 52).

Average mean frequency (0, never to 10, very often) of reported mobile app use for clinical care, overall, was 6.38 (*n* = 92), and higher for physicians (mean 6.50, *n* = 28) than nurses (mean 6.33, *n* = 64). Average mean frequency of mobile app use for personal reasons, overall, was higher than app use for clinical care (overall mean 7.65, *n* = 92), and was higher for physicians (mean 8.14, *n* = 28) compared to nurses (mean 7.44, *n* = 64).

In total, 90 respondents (*n* = 90; 93%) answered the final survey item (the optional free-text question, ‘Is there anything else you would like to share about your experience with cancer pain in Nepal?’). Respondents shared key challenges related to managing cancer pain in Nepal, and also offered suggestions for improvement. Responses focused on four overarching themes, including: access to care; cancer pain as common and challenging; need to improve training and awareness; and concerns related to opioids. Table 9 presents the key themes along with supporting exemplar responses.

**Table 9:** Summary of free-text survey responses related to managing cancer pain in Nepal

Theme	Exemplar Survey Responses
Access to Palliative Care Services	It would be better if palliative care services could be made accessible in rural areas of Nepal as well. [There is] no expert team outside the [Kathmandu] valley, medicines (morphine and other narcotics) are not easily available outside the valley, no palliative care is provided outside the valley.
Cancer Pain is Common and Challenging	Cancer pain management is one of the challenging conditions for the palliative care worker. 70% of patients have some sort of pain. But most don’t speak about it, they only talk about it if asked. In Nepal the cancer patient are more than other countries. Poor people cannot afford to be checked by a doctor. And then some people die due to lack of money and lack of knowledge and due to disease condition.
Training and Awareness of Palliative Care	Education regarding cancer and pain in cancer must be provided to all the specialties. Pain management is poor in our country not due to patients’ ignorance but lack of awareness among health care providers. I’m working in palliative care center since 16 years but lot of Nepali people (health workers) have no knowledge about the service. Need quality training. [Patients in pain] are unmanaged at home so must train lower level health worker for community service [in palliative care]. More education and knowledge for patient and caregiver about pain. We need sensitizing course not only for nurses and doctors, but for all staff. Nepalese people believe that cancer pain is untreatable. Most of the cancer patient with pain think that morphine causes addiction. So, I think there is need of more awareness regarding cancer pain management through various workshops and training programs in rural areas too.
Concerns Related to Opioids	In our palliative care unit patient come with severe pain to worse pain. Some patients worry to take morphine and refuse to take. Morphine not easily available in every health sector and fentanyl also not available and more expensive. Health care providers have knowledge but cannot apply in practice. Prescription pad/paper are not easily available. In Nepal there is very low use of opioids analgesics like morphine due to fear of respiratory depression which is very less (only 1% of cases) so training to the doctors and nurses regarding this should be done. Last few years, cancer pain management has improved in our country with the availability of access of drugs like morphine. As patient present most of the times in advance stage our focus to provide quality of life has improved with better pain management.

## Discussion

Our survey results provide important insights into cancer pain management in Nepal and make a unique contribution to inform design of a mobile app to support implementation of locally developed PMG. To our knowledge this is the first report that identifies barriers to cancer pain management and smart phone use within private sector and government/public sector hospitals, from the perspective of both nurses and physicians within an LMIC. A strength of this work is the participatory approach, which is considered essential to improve palliative care delivery in LMICs [24].

Overall, our sample reflects a subset of predominantly young healthcare providers who spend a high proportion of their clinical work caring for patients with cancer. The majority of respondents were nurses (at all sites more nurses are employed than physicians) and female, consistent with global gender demographics of nurses [47]. Statistically significant differences were detected despite the small sample sizes of some cells, suggesting that these differences may be particularly relevant.

### Confidence, knowledge and attitudes related to cancer pain management

Our overall findings related to general opioid confidence, knowledge and beliefs are encouraging. Across all sites, providers rated average helpfulness of palliative care/cancer pain management training as high (all at least a 7 on a 0–10 scale) and this rating was statistically significantly higher, overall, for nurses compared to physicians. This finding underscores the importance of ensuring interdisciplinary engagement in the creation and implementation of palliative care training content [19, 48–51].

Variations in confidence levels related to specific cancer pain management tasks were seen across institutions and by roles (e.g., nurses within the public cancer hospital reported significantly higher confidence levels in calculating breakthrough doses of morphine and adjusting/titrating morphine doses compared to physicians). Reasons for variation across institutions and roles are likely related to multiple factors, including previous palliative care training (e.g., we found a higher percentage of nurses, overall, completed training in palliative care/cancer pain management compared to physicians), autonomy and scope of practice expectations within the specific institution (e.g., public sector hospital nurses may have more autonomy than nurses in private sector hospitals in terms of opioid dosing and administration [19, 48]), and experience and exposure to cancer patients in pain (e.g., providers in cancer-specific hospitals are likely to have more regular interactions with cancer patients in pain). These findings emphasize the importance of understanding that clinical expectations and expertise may vary across site and role and the need to account for this reality in designing clinical care mobile apps. For example, a user could tailor the app to emphasize different support needed by starring most useful/needed features and ‘hiding’ information that is less useful or pertinent.

Across all study sites both physicians and nurses endorsed that cancer pain is difficult but can usually be controlled and strongly agreed that it is their role to manage cancer pain. Additionally, providers perceived morphine to be a beneficial and helpful medication, and not only for patients who are actively dying. Both nurses and physicians at the public general hospital most accurately agreed that regular morphine use will cause physical dependence, however other respondents largely disagreed, which could have worrying patient care implications, especially for opioid withdrawal. Most respondents disagreed that morphine use in cancer patients commonly causes respiratory depression; this knowledge could prevent concern over this side effect leading to a failure to prescribe morphine, but conversely care is also needed as respiratory depression is possible if safe prescribing practices are not followed. Collectively, these findings suggest information within pain management apps related to opioid pharmacology (e.g., physical dependence, respiratory depression) is likely always important to include to support busy healthcare professionals and prevent patient harm – even for users with prior palliative care training.

### Opioid availability and reported approaches to cancer pain management

Lack of opioid availability is a serious concern in the delivery of palliative care and can be especially difficult in LMICs [6]. However, opioid availability was not reported to be a primary concern in our study. Morphine, tramadol and codeine were generally available at the study sites, with few reported shortages of short duration. This is a very encouraging finding and validates Nepal’s participation in the International Pain Policy Fellowship program, which helped facilitate the initiation of in-country manufacturing of additional formulations of morphine [16]. However, our free text responses indicated limited opioid availability in rural areas and less reliable availability of fentanyl due to its increased cost. These findings suggest that focusing mobile app support on the most commonly used and available formulations of opioids (e.g., morphine syrup) is the most effective way to support healthcare providers in pain management decisions. Additionally, our survey confirms the importance of non-opioid pharmacological therapies (e.g., NSAIDs, steroids, paracetamol, gabapentin and pre-gabalin) and non-pharmacological therapies (particularly heat/cold packs and massage) to manage cancer pain, and these treatments are also critical to include in mobile app treatment algorithms.

### Barriers to cancer pain management

Our results contribute to a more complete understanding of barriers to cancer pain management within LMICs, particularly between private and public sector hospitals which represent very different care contexts. Public/government sector hospitals generally provide subsidized care to a large volume of patients of lower socioeconomic status and must cope with significant resource constraints; private sector hospitals generally care for more financially secure patients who can pay out of pocket for treatment and operate with greater resources. This resource disparity is likely represented in our finding that respondents, regardless of role, at the private cancer hospital rated both institutional and personal barriers to cancer pain management very low compared to other hospitals and the overall ratings. These institutional differences are important to understand as they influence contextual barriers to cancer pain management, and thus, potential adherence or non-adherence to PMG.

Key institutional barriers across all sites, and for both nurses and physicians, related to the high volume of patients they care for and lack of dedicated palliative care beds or outpatient department. Mobile app design can account for these barriers to some extent by ensuring that user interfaces are as quick and efficient as possible to accommodate brief patient care interactions and include clear, concise recommendations. It is encouraging that concerns regarding addiction and repercussions for prescribing/administering opioids were not highly rated barriers. It is especially encouraging that these barriers were the lowest rated among nurses, as they are often the ones to decide whether to administer an as-needed (PRN) pain medication. We also find this encouraging as a contrast to prior research that indicates fears related to addiction and/or regulatory surveillance can limit opioid prescribing/administration, even for patients with legitimate, severe pain, in both high and low income countries [52, 53]. However, a less palliative care sensitized sample may have reported greater concerns related to addiction and consequences of prescribing/administering opioids and more supports and resources within an app may be needed for a general practice user.

Consistent with prior research [54] our findings reinforce the significant role of system-level country and cultural factors related to cancer pain management, particularly related to access to care, opioid availability, and the need for palliative care training and sensitization not only for healthcare providers and clinic/hospital staff, but community health workers and patients and family caregivers. Some country/cultural level barriers are more applicable to the design of a mobile app than others, but they are all important to consider on a macro-level to improve the delivery of palliative care within Nepal, and similar settings. For example, a highly rated country/cultural level barrier, and a key theme within our free-text responses, related to patients in rural areas who have difficulty accessing healthcare [55]. Creating cancer pain management apps not dependent on consistent internet service that can be used by community health workers in rural areas is one way to address this barrier. Additionally, while healthcare providers themselves may not fear causing opioid-related addiction, they (especially nurses) do feel this is a significant concern for patients and their families. This finding emphasizes the importance of an app that has an educational component or companion features to specifically support patients and family members, or that offers healthcare users guidance with tools and language to educate patients and family members regarding common opioid-related concerns, such as stigma, respiratory depression or addiction.

### Awareness and use of the NAPCare PMG

It is encouraging that such a large proportion of respondents had heard of the NAPCare PMG, reported using them multiple times a day, and strongly agreed that the guidelines were helpful in their day-to-day clinical practice. We are optimistic that such a degree of pre-existing awareness and receptivity to the NAPCare PMG bodes well for use of a mobile app. It is also validating that the most frequently used ‘other’ cancer pain management guideline by participants were the WHO guidelines, since they serve as the foundation for the NAPCare PMG.

### Smart phone usage and desired features and evaluation metrics of the mobile app

Our survey confirms the ubiquity of smart phones in LMICs [34] and the frequent use of apps for both personal and professional reasons. Importantly, our results also highlight unique disciplinary barriers to smart phone use. The vast majority of physicians indicated they encountered no barriers in using smart phones, whereas this was not true for nurses. The cost of smart phones and data plans were significant barriers for a larger percentage of nurses than physicians, and many nurses reported they are prohibited from using smart phones at work by their employer, a restriction that does not apply to most physicians. Nurses may need additional supports to utilize mobile apps to enhance clinical care, such as financial assistance to purchase phones or data plans, the use of institution-owned mobile phones during their shift, or approval by their immediate supervisor to use their personal mobile phone to support the delivery of patient care.

Overall, and especially for nurses, the most desired feature to better manage cancer pain was the ability of the app to educate patients and family members. While this feature is not within scope for the first iteration of our app, it is clearly a critical future component to include. Regarding evaluation of the app, key user-defined metrics include frequency of app use; confidence levels in prescribing/administering pain therapies; and adherence to the NAPCare PMG, including documenting reasons when PMG cannot be followed (e.g., preferred medication is out of stock) to help inform future tailored interventions.

#### Limitations

The primary limitation of this study is the convenience (versus probability-based) sample of palliative care sensitized healthcare providers and the respondent quotas we established prior to survey administration. Our sample size also limited our ability to conduct more detailed multivariate analyses to more robustly explore significant differences among groups; this would be an important area of future research. However, our sampling strategy was in keeping with the pilot nature of this grant and the overall intention to mentor, and not overburden, new investigators within Nepal. In hindsight we underestimated the number of participants willing to complete our survey, and could have likely surveyed a larger, more diverse sample of clinicians, particularly at the public general and cancer hospitals. As with any survey, there is the risk of response and recall bias; we attempted to mitigate this by taking care to write our questions in neutral, clear language and by providing both remote and in-person training and support to Nepal team members who administered the survey.

#### Implications and recommendations

In this paper we describe a survey designed to identify gaps and opportunities in cancer pain management in Nepal with a focus on informing design of a tailored digital health intervention to support clinicians providing care to patients with cancer. However, it is important to note that any digital health intervention can have unintended consequences [56], such as diverting provider attention from the actual patient or supplanting clinical assessment. Apps to help manage cancer pain within Nepal, or any clinical setting, are meant to be supportive adjuncts and not intended to replace training, authentic patient engagement, or expert clinical judgment. Additionally, our survey highlights critical system-level barriers to cancer pain management, such as the need for improved healthcare infrastructure in rural areas, quality generalist palliative care training and sensitization across all levels of the healthcare sector, and continued vigilance to ensure opioid availability and mitigate opioid-phobia that can derail patient comfort. Of course, no mobile app can fully address all of these barriers; however, digital health interventions, such as the NAPCare PMG app, are one important tool in our armamentarium to help promote culturally and resource-congruent support to healthcare providers and patients in LMICs. Future work includes scaling-up design and testing the NAPCare PMG app on relevant patient, provider and institutional outcomes.

## Conclusion

Our survey of diverse cancer care institutions within Nepal emphasizes that healthcare providers view cancer pain as an important symptom management concern, use smart phones and apps frequently, and are receptive to a mobile app to provide PMG support. Mobile apps must be informed by a clear understanding of contextual barriers to both cancer pain management and smart phone usage that are influenced by institutional resource and disciplinary differences.

## Data Availability

The datasets generated and analyzed are not publicly available due to participant privacy concerns, but de-identified, aggregated data are available from the corresponding author upon reasonable request and in compliance with institutional data sharing protocols.
